# Methanolic Extract of *Micromeria frivaldszkyana* (Degen) Velen Alleviates Tert-Butyl Hydroperoxide-Induced Hepatic Damage and Renal Function-Related Serum Biomarkers in Male Wistar Rats

**DOI:** 10.3390/cimb48070646

**Published:** 2026-06-23

**Authors:** Kristina Stavrakeva, Elisaveta Apostolova, Vesela Kokova, Ivica Dimov, Mariya Choneva, Delyan Delev, Ilia Kostadinov, Ilia Bivolarski, Maria Koleva, Rumen Mladenov, Plamen Stoyanov, Anelia Bivolarska

**Affiliations:** 1 Department of Pharmacology, Toxicology, and Pharmacotherapy, Faculty of Pharmacy, Medical University of Plovdiv, Vasil Aprilov Str. 15A, 4002 Plovdiv, Bulgaria; elisaveta.apostolova@mu-plovdiv.bg (E.A.); vesela.kokova@mu-plovdiv.bg (V.K.); 2 Research Institute, Medical University of Plovdiv, 4002 Plovdiv, Bulgaria; iliya.kostadinov@mu-plovdiv.bg; 3 Center for Competence “PERIMED-2”, Vasil Aprilov Blvd. 15A, 4002 Plovdiv, Bulgaria; 4 Department of Medical Biochemistry, Faculty of Pharmacy, Medical University of Plovdiv, Vasil Aprilov Str. 15A, 4002 Plovdiv, Bulgaria; ivica.dimov@mu-plovdiv.bg (I.D.); mariya.choneva@mu-plovdiv.bg (M.C.); anelia.bivolarska@mu-plovdiv.bg (A.B.); 5 Department of Pharmacology and Clinical Pharmacology, Faculty of Medicine, Medical University of Plovdiv, Vasil Aprilov Str. 15A, 4002 Plovdiv, Bulgaria; delyan.delev@mu-plovdiv.bg; 6 Department of General and Clinical Pathology, Faculty of Medicine, Medical University of Plovdiv, 4000 Plovdiv, Bulgaria; iliya.bivolarski@mu-plovdiv.bg (I.B.); mariya.koleva@mu-plovdiv.bg (M.K.); 7 Department of Botany and Biological Education, Faculty of Biology, University of Plovdiv “Paisii Hilendarski”, 24 Tsar Assen Str., 4000 Plovdiv, Bulgaria; rummlad@uni-plovdiv.bg (R.M.); pstoyanov@uni-plovdiv.bg (P.S.); 8 Department of Bioorganic Chemistry, Faculty of Pharmacy, Medical University of Plovdiv, Vasil Aprilov Str. 15A, 4002 Plovdiv, Bulgaria

**Keywords:** *Micromeria frivaldszkyana*, methanolic extract, t-BHP, hepatotoxicity, oxidative stress, Wistar rats, biochemical markers, rosmarinic acid, histopathology

## Abstract

Plant-derived compounds have recently attracted considerable scientific attention due to their potential therapeutic applications, which are largely attributed to their antioxidant properties. Tert-butyl hydroperoxide (t-BHP) is a potent inducer of intracellular oxidative stress, generating reactive free radicals, which significantly contribute to hepatic and renal damage. *Micromeria frivaldszkyana* (*M. frivaldszkyana*), a Bulgarian endemic species, contains high levels of phenolic compounds, including linarin, rosmarinic acid (RA), chlorogenic acid, rutin, quercetin, naringenin, and apigenin. In this study, male Wistar rats received oral treatment for 5 days comprising saline, 250, 400, or 500 mg/kg of *M. frivaldszkyana* methanolic extract, 100 mg/kg RA, or 125 mg/kg silymarin. On the final day, 0.5 mmol/kg of t-BHP was injected intraperitoneally, and blood and liver tissue samples were collected 18 h later for biochemical and histological analysis. Liver and kidney function was evaluated using biochemical markers (alanine aminotransferase (ALT), aspartate aminotransferase (AST), urea, creatinine (Cr), uric acid (UA)), indicators of oxidative stress (malondialdehyde (MDA), 8-hydroxy-2′-deoxyguanosine (8-OHdG), glutathione (GSH), superoxide dismutase (SOD), catalase (CAT)), and histopathology. Exposure to t-BHP resulted in significant hepatic and renal damage, including elevated serum markers, increased lipid peroxidation, and deoxyribonucleic acid (DNA) damage. Administration of 500 mg/kg *M. frivaldszkyana* markedly lowered elevated serum ALT and AST levels. The extract also significantly mitigated t-BHP-induced increases in serum Cr and UA. However, no significant increase in the levels of the antioxidant enzymes SOD and CAT or in GSH was observed at all tested doses. Malondialdehyde and 8-OHdG levels increased markedly following t-BHP exposure, whereas pretreatment with *M. frivaldszkyana* at all tested doses significantly ameliorated these oxidative alterations. These findings suggest that the methanolic extract of *M. frivaldszkyana* confers protective effects against t-BHP-induced toxicity, potentially through stabilisation of cell membranes, inhibition of lipid peroxidation, and reduction in DNA damage. The extract may therefore serve as a potential natural therapeutic agent against injuries caused by oxidative stress.

## 1. Introduction

Hepatic injury can be caused by various etiological factors, including viral infections, alcohol consumption, pharmaceutical agents, and disturbances in bile synthesis and secretion. The underlying mechanisms of hepatocellular damage are highly diverse and form the foundation for developing experimental models of hepatotoxicity in laboratory animals [1]. Paracetamol (acetaminophen) is one of the most frequently used agents to induce experimental liver injury, while other forms of drug-induced hepatotoxicity are associated with antibiotics, antiepileptic agents (e.g., valproates), antineoplastic drugs (e.g., methotrexate), and non-steroidal anti-inflammatory drugs [2,3].

Nephrotoxicity refers to the impairment of kidney function caused by endogenous or exogenous toxic agents, including certain drugs and harmful substances. This condition compromises the kidneys’ capacity to detoxify the body effectively and eliminate metabolic waste [4]. Chronic kidney disease can be induced experimentally through the administration of antitumour drugs such as doxorubicin, cisplatin, cyclophosphamide, and methotrexate; antibiotics such as gentamicin and vancomycin; and other agents such as cyclosporine and aristolochic acid [4,5].

Tert-butyl hydroperoxide (t-BHP) is an organic hydroperoxide which, upon metabolic conversion, produces reactive free radicals and acts as a significant driver of intracellular oxidative stress [6,7]. The latter is a major contributor to the development of toxic liver injury. Tert-butyl hydroperoxide is metabolised via two pathways. The first of these involves cytochrome P450 (CYP 450), resulting in the formation of t-butoxyl and t-methyl radicals. The latter trigger lipid peroxidation of membrane phospholipids, which consequently alters membrane fluidity and permeability. The second pathway is mediated by glutathione peroxidase (GPx) and results in the detoxification of t-BHP to tert-butanol. This process contributes to the depletion of glutathione (GSH) [8,9]. The antioxidant potential of various compounds in different in vitro and in vivo models is commonly evaluated by inducing oxidative damage in hepatocytes through exposure to t-BHP [6,7].

Excessive oxidative stress may result from the excessive generation of reactive oxygen species (ROS), which contribute to the onset and progression of chronic kidney disease [10]. Malondialdehyde (MDA), a terminal product of lipid peroxidation, is widely recognised as a biomarker of oxidative stress. Antioxidant enzymes, including catalase (CAT) and superoxide dismutase (SOD), play a crucial role in reducing oxidative damage [11]. Moreover, GSH constitutes an essential protective mechanism by scavenging ROS and preserving tissue integrity against oxidative injury. Urea and creatinine (Cr) levels serve as key diagnostic indicators used to assess renal function and structural integrity, while serum levels of alanine aminotransferase (ALT) and aspartate aminotransferase (AST) are commonly used to evaluate liver function and detect hepatic disorders [12]. Eight-hydroxy-2′-deoxyguanosine (8-OHdG) is used as a biomarker of hepatocyte deoxyribonucleic acid (DNA) injury. Hydroxyl radicals, generated by oxidants, damage genetic material, whereas prior administration of free radical scavengers can decrease oxidative stress and DNA disruption [13]. Cellular damage can be prevented by enhancing cells’ antioxidant defence mechanisms through the administration of antioxidants. In recent years, considerable attention has been directed toward the beneficial effects of naturally occurring antioxidants found in plants, fungi, and algae.

Plant-derived phenolic phytochemicals are increasingly recognised for their potential and are widely used in the prevention and treatment of conditions related to oxidative stress. The protective effects of these compounds are largely attributed to the antioxidant and free radical scavenging activities of their constituent polyphenols and flavonoids [14]. Silymarin, a standardised flavonolignan mixture derived from milk thistle (*Silybum marianum*), was used in this study as the standard reference due to its well-established antioxidant and hepatoprotective properties [15,16]. Isolated phytochemicals [17,18], plant extracts [19,20,21], and certain fungi, e.g., *Pleurotus ostreatus* [22], have been proposed in the scientific literature as nephroprotective agents.

*Micromeria frivaldszkyana* (*M. frivaldszkyana*) is a Bulgarian endemic species belonging to the Lamiaceae family. It is classified as endangered in the Red Data Book of the Republic of Bulgaria [23,24]. Due to its restricted natural distribution, research on this species is limited. Initial investigations of *M. frivaldszkyana* primarily focused on its phytochemical composition [25,26]. The in vitro antioxidant potential of *M. frivaldszkyana* was assessed using six assays—2,2-diphenyl-1-picrylhydrazyl, 2,2-azinobis-(3)-ethylbenzthiazoline-6-sulfonic acid, ferric reducing antioxidant power, cupric ion reducing antioxidant capacity, oxygen radical absorbance capacity (ORAC), and hydroxyl radical averting capacity—revealing notably high activity, with the ORAC value exceeding that of many other Bulgarian medicinal plants [27]. Subsequent in vivo studies demonstrated that the oral administration of methanolic plant extract, both as a single dose of up to 5000 mg/kg body weight and as a 90-day treatment at 250 mg/kg and 500 mg/kg, caused no mortality or observable toxic effects in male Wistar rats [28,29]. Moreover, the methanolic extract has demonstrated hepatoprotective and nephroprotective potential in models of acetaminophen-induced liver and kidney damage in the same rat species [30,31].

Given the different mechanisms underlying the toxicity of commonly used hepato- and nephrotoxic agents, as well as the limited knowledge regarding the biological activity of the studied plant species, the present study aimed to evaluate the effects of orally administered *M. frivaldszkyana* methanolic extract on t-BHP-induced liver injury and changes in serum renal function biomarkers in rats. The severity of organ injury was studied through histopathological examination and measurement of specific biochemical markers in serum and organ tissues.

## 2. Materials and Methods

The experimental protocol was approved by the Bulgarian Food Safety Agency (permit No. 352/30 May 2023) and the Ethics Committee of the Medical University–Plovdiv (protocol No. 6/5 October 2023). Collection of plant material was authorised by the Ministry of Environment and Water (permit No. 996/9 August 2023). The entire study was conducted in compliance with the ARRIVE guidelines, the EU Directive 2010/63/EU on animal experiments, and all relevant national and institutional regulations.

### 2.1. Chemicals and Reagents

Methanol (≥99.8%, cat. No. 179337), RA (≥96.0%, batch No. BCCJ6033), t-BHP (70% water solution batch No. MKCR8130), and silymarin (≥30.0%, batch No. BCCH4151) were purchased from Merck SA (Darmstadt, Germany). Eosin Y (1% aqueous solution, cat. No. 294/EOY-10-OT-2.5L), formaldehyde 4% (10% neutral buffer, cat. No.294/FNB4-10L), Histanol 100 (cat. No.294/H100-5L), Histanol 95 (cat. No. 294/H95-5L), haematoxylin G3 (cat. No. 294/HEMG3-OT-2.5L), acetone (cat. No.48/3413/5) and xylol (cat. No. 348/3410/20) were purchased from BIOCARE Medical (Pacheco, CA, USA).

### 2.2. Plant Material and Preparation of the Methanolic Extract

Approximately 800 g of fresh aerial parts of *M. frivaldszkyana* was obtained during the 2023–2024 vegetation period in the Bulgarka Nature Park, situated in the Middle Stara Planina floristic region, based on previously established procedures [28,29,30]. A voucher specimen of *M. frivaldszkyana* is preserved in the herbarium of the Agricultural University–Plovdiv (SOA) under accession number 062648. The methanolic extract used in the present animal study was the same batch as that used in our previous study [28].

The plant material was air-dried for 10 days in a shaded environment at 22 ± 2 °C. Complete drying was achieved when the leaf stalks split under bending and the petioles disintegrated easily. The dried samples were subsequently pulverised into a fine powder, with particles smaller than 400 µm, using a GRINDOMIX GM200 laboratory mill (RETSCH GmbH, Haan, Germany) and stored in paper bags prior to extraction.

The extraction process involved macerating 10 g of powdered plant material in 70% methanol (1:10 *w*/*v*) with constant stirring for 24 h at room temperature (22 ± 2 °C) in a light-shielded vessel. To improve extraction performance, the mixture was exposed to three consecutive 15 min ultrasonication treatments at 30 °C. The obtained extract was afterwards centrifuged at 6000× *g* for 15 min on an MPW-352R centrifuge (MPW Med. Instruments, Warsaw, Poland), and the resulting supernatant was filtered through Whatman No. 1 filter paper (Sigma-Aldrich, Burlington, MA, USA) to remove particulates.

The remaining plant residue underwent the same extraction procedure twice more. The combined extracts were concentrated to dryness using a Heidolph rotary evaporator (Schwabach, Germany) at 50 °C under reduced pressure. This process yielded 5.48 g of dried extract, representing 54.8% of the original dry plant material.

### 2.3. Animals and Treatment

Fifty-six male Wistar rats weighing 130–280 g were used in the study and randomly divided into eight groups of seven animals each. The animals were housed in standard laboratory settings, at 22 ± 1 °C, 45% humidity, a 12:12 h light/dark cycle, and free access to food and water. Each group was kept in a separate plastic cage.

Experiments were performed in the laboratory of the Department of Pharmacology and Clinical Pharmacology, Faculty of Medicine, Medical University of Plovdiv, after a one-week acclimatisation period.

The experiment was conducted according to the protocol for t-BHP-induced hepatic injury, as described by Yang et al. (2013) [32]. The rats received oral treatment for 5 consecutive days according to the following group allocation:

Groups 1 (S, control) and 3 (S+t-BHP) were administered saline at a dose of 0.1 mL/100 g body weight (bw); group 2 (ME500) received 500 mg/kg of an aqueous solution of the dried methanolic extract; groups 4, 5, and 6 (ME250+t-BHP, ME400+t-BHP, ME500+t-BHP) were treated with 250, 400, and 500 mg/kg water solution of the dried methanolic extract, respectively; group 7 (RA+t-BHP) was given 100 mg/kg RA; and group 8 (Sil+t-BHP) was treated with 125 mg/kg silymarin.

On the fifth day, 30 min after the assigned substances were administered, animals from the S+t-BHP, ME250+t-BHP, ME400+t-BHP, ME500+t-BHP, RA+t-BHP, and Sil+t-BHP groups were injected intraperitoneally with 0.5 mmol/kg t-BHP. Eighteen hours after the t-BHP injection, blood and liver samples were collected. Portions of each organ were fixed for histopathological analysis, while the remaining tissue was stored at −80 °C and later homogenised for biochemical evaluation.

The selection of doses and the duration of pretreatment before t-BHP exposure were determined according to data reported in previous research [6,32]. The methanolic extract doses were chosen following the recommendations of Hanafy et al., who proposed the usage of 1/10 and 1/20 of the estimated LD_50_ for pharmacological evaluations [33]. In our earlier work, we confirmed the safety of *M. frivaldszkyana* extract at single oral doses of up to 5000 mg/kg, which supports the dose range employed in this study [28]. To further examine the extract’s dose-related activity, an intermediate dose of 400 mg/kg was also tested.

### 2.4. Histopathological Observation

The experimental protocol was based on the descriptions of other authors [34,35,36] and was reported previously [31]. Immediately following collection, the organs were placed in a 10% neutral-buffered formalin solution to ensure proper fixation. The fixed samples were subsequently processed and embedded in paraffin blocks. The entire histological preparation included the following steps:

1. The tissues were immersed in formalin for 24 h, thoroughly rinsed, and then soaked in distilled water for 30 min to eliminate any residual fixative.
2. Dehydration was achieved by progressively immersing the sample in 95% and then 100% ethanol (Histanol^®^ 100%, BioGnost, Zagreb, Croatia), with each step lasting 4–5 h.
3. After dehydration, the tissues were treated with acetone for 20 min, followed by xylol for 30 min. They were then embedded in molten paraffin and formed into blocks. Thin 5 μm slices were obtained from these blocks and stained with haematoxylin and eosin for microscopic examination.

Histopathological analysis was performed independently by two pathologists who were blinded to the experimental groups. Morphological alterations in the tissues of the treated groups were compared to those of controls. Leica DM500 (Leica Microsystems, Wetzlar, Germany) and Zeiss AXIO Scope A1 microscopes (Carl Zeiss Microscopy GmbH, Jena, Germany) were used for sample observation.

Liver samples were examined for pathological changes such as architectural disruption, portal and lobular inflammation, sinusoidal dilation and congestion, granuloma formation, necrosis, degeneration, and steatosis. Tissue pathology was classified according to the Roenigk scoring system (Table 1).

Grading criteria—All liver specimens obtained from euthanised rats were examined by light microscopy and graded into four categories (G1, G2, G3a, G3b, and G4) using a grading system, as described by Roenigk classification (adopted from Berends et al. (2007) [37]. We added a G0 category for normal histology.

Grade 0—normal histology, characterised by preserved liver architecture; no evidence of fibrosis, sinusoidal dilation, nuclear pleomorphism, or inflammatory infiltration was observed.

Grade 1—mild toxicity;

Grade 2—moderate toxicity;

Grade 3a—severe toxicity;

Grade 3b or 4—indication for discontinuation of the treatment.

### 2.5. Preparation of Liver Homogenates and Assessment of Tissue Toxicity Markers

On the day of analysis, frozen liver samples were thawed to room temperature and homogenised in 0.1 M PBS containing Triton 100× (1:9 *w*/*v*), pH 7.4 [38], using a Polytron mechanical homogeniser (KINEMATICA, Malters, Switzerland). The resulting homogenates were then centrifuged at 10,000 rpm for 10 min at 4 °C in an MPW-352R refrigerated centrifuge (Warsaw, Poland) [39]. The obtained supernatant was collected for the evaluation of oxidative damage and antioxidant defence markers, including MDA, 8-OHdG, CAT, SOD1, and GSH.

All parameters were quantified by enzyme-linked immunosorbent assay (ELISA) using a microplate reader (HumanReader, HUMAN, Wiesbaden, Germany) and commercial assay kits [CAT ELISA Kit, Cloud-Clone Corp., Katy, TX, USA, Cat. No. SEC418Ra; MDA ELISA Kit, Cat. No. E-EL-0060; 8-OHdG ELISA Kit, Cat. No. E-EL-0028; SOD1 ELISA Kit, Cat. No. E-EL-R1424; GSH ELISA Kit, Cat. No. E-EL-0026, Elabscience Biotechnology Inc., Houston, TX, USA]. To minimise variability between batches, all kits were sourced from the same production lot, and each plate included internal controls provided by the manufacturer to ensure the reliability of the assay.

### 2.6. Biochemical Markers

Blood samples (3–4 mL) were collected in VACUTEST KIMA tubes (Arzergrande, Italy) containing a coagulation-accelerating agent and kept at refrigerated conditions until clot formation was observed. The samples were then centrifuged at 3000 rpm for 10 min at 4 °C using a refrigerated centrifuge (MPW-352R, MPW Med. Instruments, Warsaw, Poland). The separated serum was aliquoted and stored at −80 °C (ULT C200) until analysis.

Serum samples were analysed spectrophotometrically to determine markers of liver function—AST, ALT, total bilirubin, conjugated bilirubin, and kidney function indicators, including urea, Cr, and UA. Measurements were performed on an Evolution 300 UV-Vis spectrophotometer (Thermo Fisher Scientific, Waltham, MA, USA) following the manufacturer’s protocols provided with the corresponding commercial kits (HUMAN Diagnostics GmbH, Wiesbaden, Germany).

### 2.7. Statistical Analysis

Statistical analyses were performed using SPSS software, version 17.0 (IBM, Armonk, NY, USA). Data are expressed as mean ± SEM. Group differences were assessed by one-way analysis of variance (ANOVA) followed by Tukey’s post hoc test. Histological scores based on the Roenigk classification were converted into ordinal numerical values (G0 = 0, G1 = 1, G2 = 2, G3a = 3) and analysed using the Kruskal–Wallis test. A *p*-value of ≤0.05 was considered statistically significant. The number of tested animals is given as *n*.

## 3. Results

### 3.1. Effect of Methanolic Extract of M. frivaldszkyana in t-BHP-Induced Liver Toxicity

**Figure 1:**
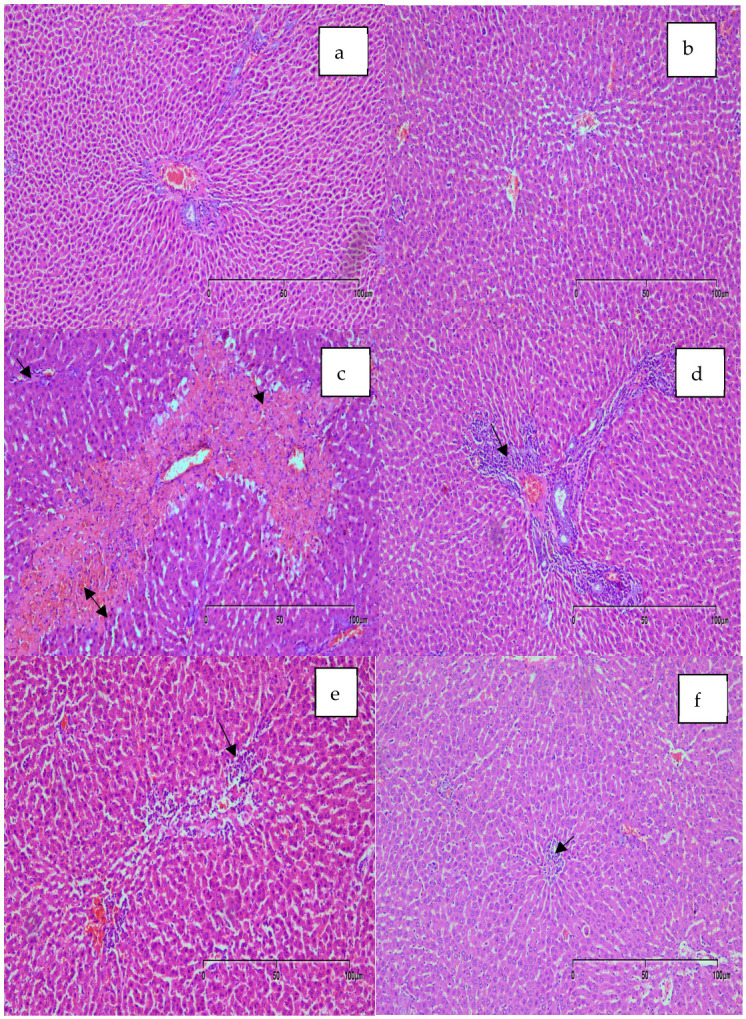

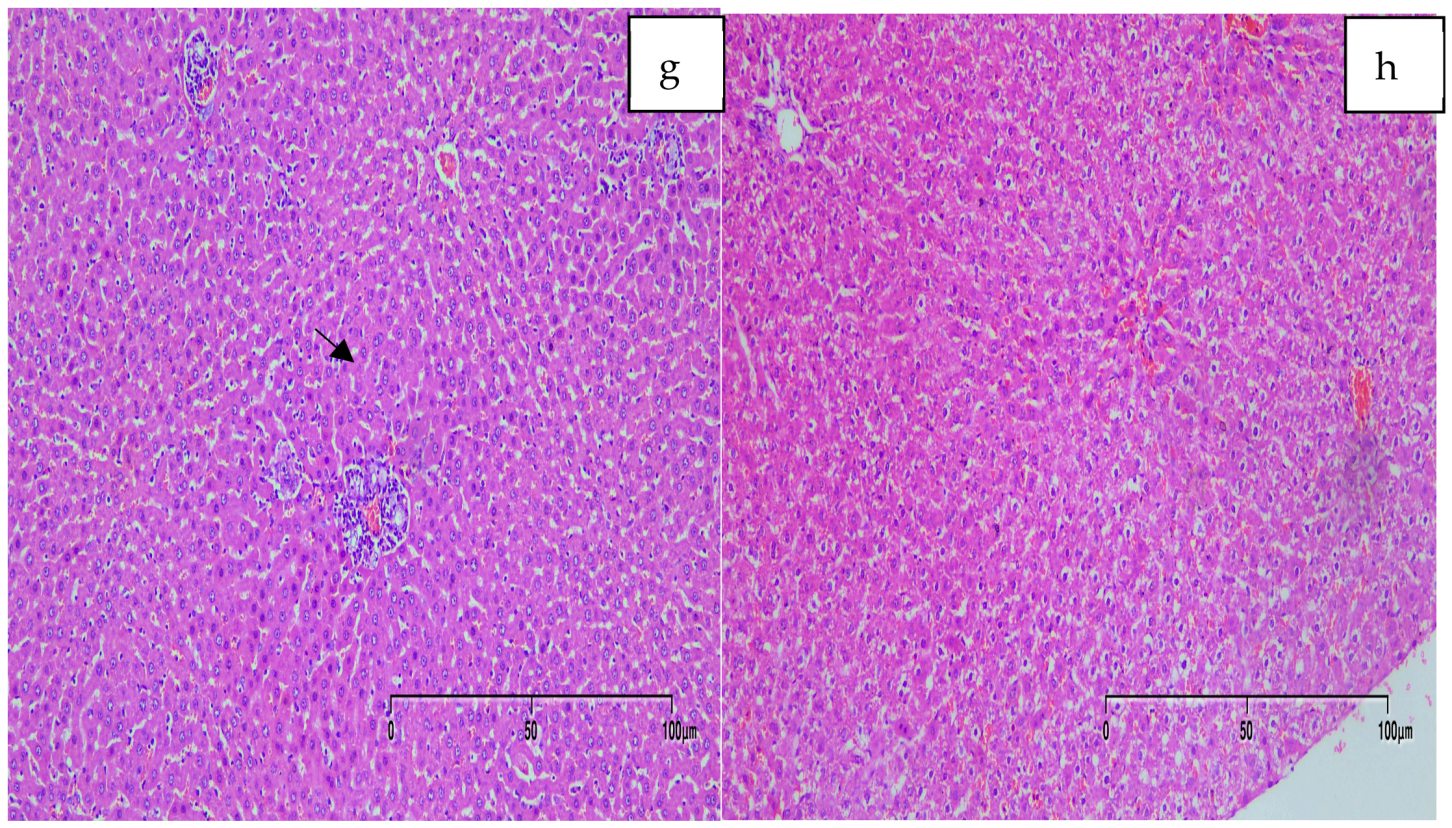
Histological examination of liver tissue. (**a**) Group S—control rats, treated with saline at 0.1 mL/100 g bw without t-BHP application; (**b**) group ME500 received 500 mg/kg of a water solution of the dried methanolic extract without t-BHP application; (**c**) group 3 (S+t-BHP) received 0.1 mL/100 g saline and t-BHP application; (**d**) group 4 (ME250+t-BHP) received 250 mg/kg water solution of the dried methanolic extract and t-BHP application; (**e**) group 5 (ME400+t-BHP) rats were treated with a water solution of the dried methanolic extract at a dose of 400 mg/kg and t-BHP application; (**f**) group 6 (ME500+t-BHP) received a water solution of the dried methanolic extract at a dose of 500 mg/kg and t-BHP application; (**g**) group 7 (RA+t-BHP) was treated with 100 mg/kg RA and t-BHP application; (**h**) group 8 (Sil+t-BHP) was treated with 125 mg/kg silymarin and t-BHP application; haematoxylin and eosin (H&E) ×100.

As shown in Figure 1, normal liver architecture was observed in the control rats (Figure 1a) and ME500 group (Figure 1b). According to the Roenigk classification (adopted from Berends et al. (2007) [37], which categorises toxicity as mild (G1), moderate (G2), severe (G3a), or Grade 3b or 4 (indicating discontinuation of treatment), the most significant pathological changes were detected in the S+t-BHP group (*H* = 22.02, *p* = 0.00052) (Figure 1c). In this group, fibrosis, focal nuclear pleomorphism, focal parenchymal haemorrhages and inflammatory cell infiltration predominantly consisting of lymphocytes and plasma cells were observed, as well as disruption of normal liver architecture. Treatment with the methanolic extract of *M. frivaldszkyana* (Figure 1d–f) reduced the severity of liver damage, though not to the same extent as in rats treated with silymarin (*p* = 0.001) (Figure 1h). No statistically significant improvement was observed in the ME250+t-BHP group (*p* = 0.170), while the ME400+t-BHP group showed a trend towards reduced liver injury that did not reach statistical significance (*p* = 0.062). In contrast, treatment with 500 mg/kg of the methanolic extract significantly reduced liver damage (*p* = 0.008). A reduction in liver damage was also observed in rats treated with RA (*p* = 0.034) (Figure 1g).

No pathological hepatic alterations were detected in the Control S or ME500 groups. In contrast, the S+t-BHP group displayed the most severe pathological changes. Fibrosis (short arrow), foci of nuclear pleomorphism, focal parenchymal haemorrhages (double-headed arrow) and inflammatory infiltration predominantly by lymphocytes and plasma cells (long arrow) accompanied by disruption of the normal hepatic architecture were found. In the 250 mg/kg methanolic extract + t-BHP group, no fibrosis was observed in the hepatocytes, and severe inflammatory infiltration characterised by lymphocytes and plasma cells (arrow) was present. Comparable findings were shown by the 400 mg/kg methanolic extract + t-BHP group, with no fibrosis and moderate inflammatory infiltration presented by lymphocytes and plasma cells (arrow). The 500 mg/kg methanolic extract+t-BHP group exhibited results similar to those of the silymarin group. There was a lack of fibrosis of hepatocytes and no nuclear pleomorphism. Focal mild chronic inflammatory infiltration presented by single lymphocytes was observed (arrow). In the RA+t-BHP group, no fibrosis was found, and the inflammatory infiltration present was characterised by lymphocytes and plasma cells (arrow). Only minimal pathological changes were observed in the silymarin+t-BHP group. There was no fibrosis, and the inflammatory infiltration was mild.

According to the Roenigk classification (adopted from Berends et al. (2007) [37], the following quantitative histological analysis can be performed:

Group 1—S (control group)—administered 0.1 mL/100 g body weight (bw) of saline without t-BHP application; all 8 cases (100%—G0);

Group 2—ME500—received 500 mg/kg of a water solution of the dried methanolic extract without t-BHP application; all 8 cases (100%—G0);

Group 3—S+t-BHP—received 0.1 mL/100 g saline and t-BHP application; 2 cases (25%—G3a) and 6 cases (75%—G2);

Group 4—ME250+t-BHP—received 250 mg/kg water solution of the dried methanolic extract and t-BHP application; 8 cases (100%—G2);

Group 5—ME400+t-BHP—administered a water solution of the dried methanolic extract at a dose of 400 mg/kg and t-BHP application; 2 cases (25%—G1) and 6 cases (75%—G2);

Group 6—ME500+t-BHP—administered a water solution of the dried methanolic extract at a dose of 500 mg/kg and t-BHP application; 5 cases (62.5%—G1) and 3 cases (37.5%—G2);

Group 7—RA+t-BHP—administered 100 mg/kg RA and t-BHP application; 3 cases (37.5%—G1) and 5 cases (62.5%—G2);

Group 8—Sil+t-BHP—administered 125 mg/kg silymarin and t-BHP application; 7 cases (87.5%—G1) and 1 case (12.5%—G2).

### 3.2. Changes in Serum Biomarker Levels in t-BHP-Induced Toxicity

**Figure 2:**
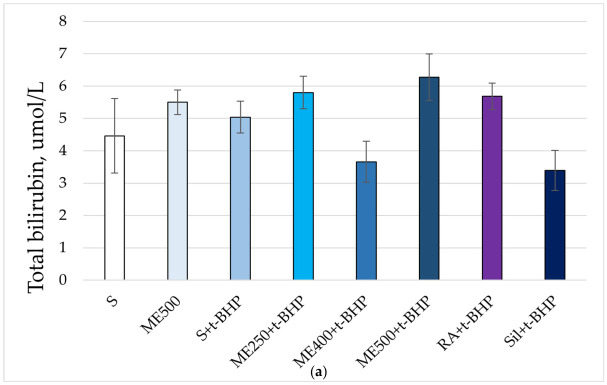

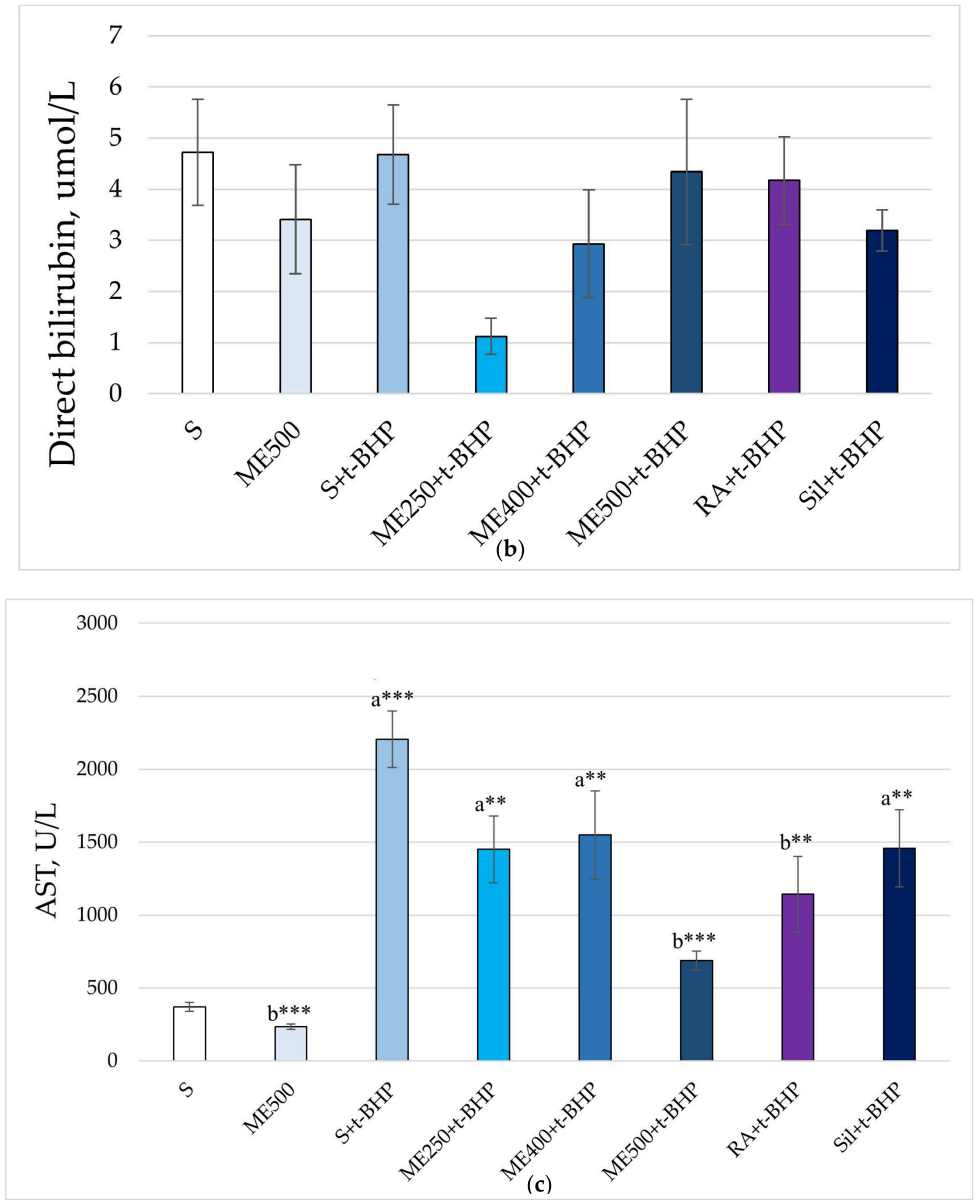

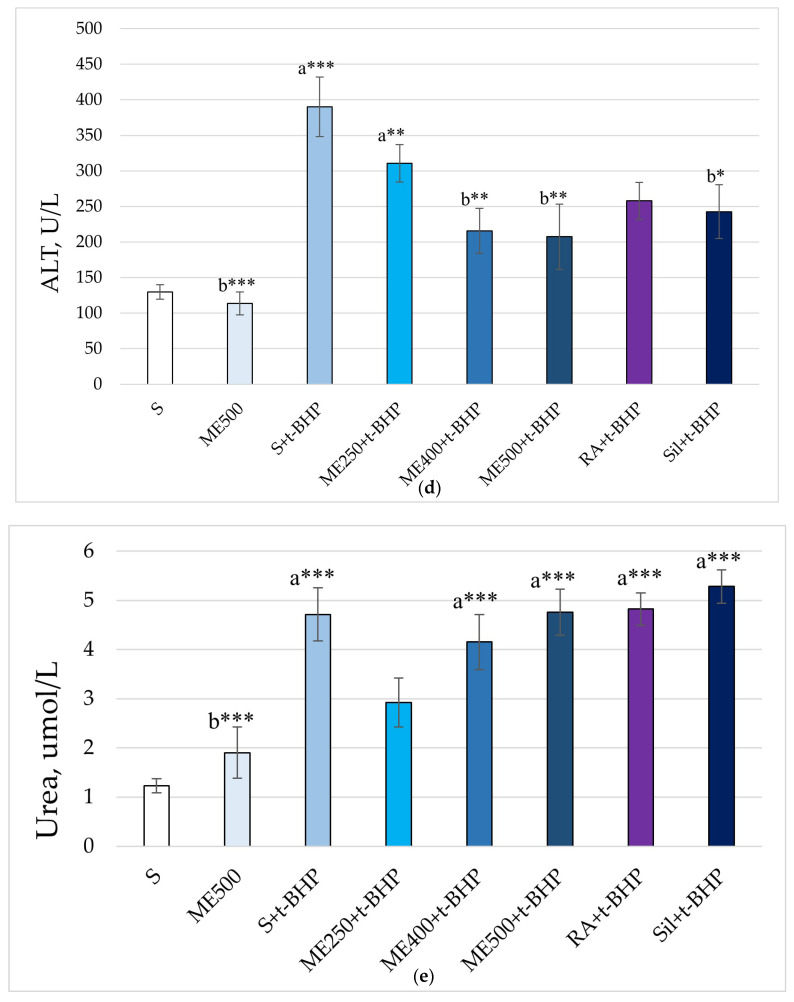

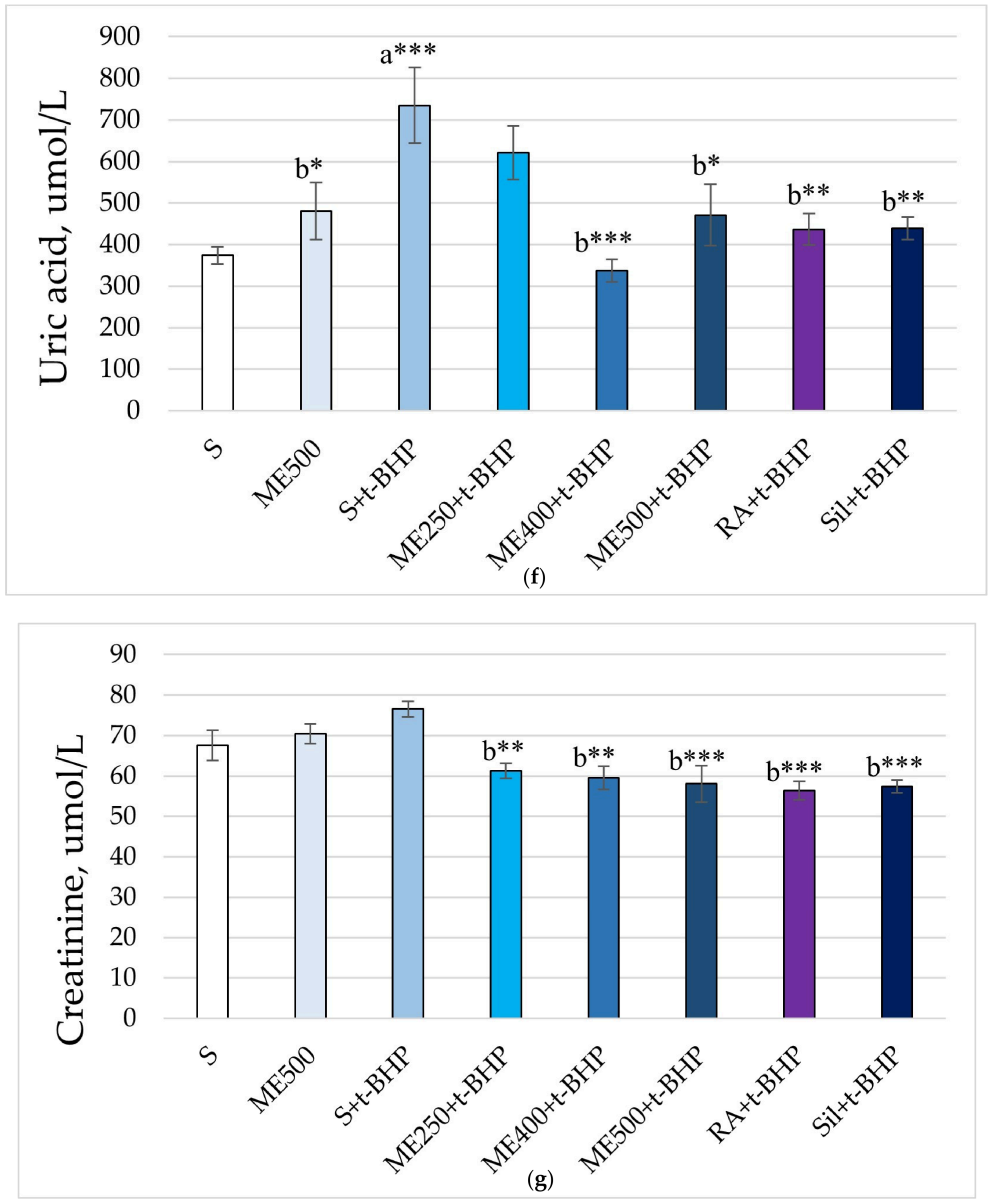
Biochemical markers in serum. (**a**) Total bilirubin; (**b**) conjugated bilirubin; (**c**) AST; (**d**) ALT; (**e**) urea; (**f**) UA; (**g**) Cr. Data are presented as mean ± SEM. The symbol a** indicates *p* < 0.01 vs. control group; a*** indicates *p* ≤ 0.001 vs. control group; b* indicates *p* ≤ 0.05 vs. t-BHP + saline-treated rats; b** indicates *p* ≤ 0.01 vs. t-BHP + saline-treated rats; b*** indicates *p* ≤ 0.001 vs. t-BHP + saline-treated rats, according to one-way ANOVA followed by Tukey’s post hoc test.

No statistically significant differences were observed in total and direct bilirubin levels, as can be seen in Figure 2a,b.

Figure 2c illustrates significantly higher AST levels in the S+t-BHP, ME250+t-BHP, ME400+t-BHP, and Sil+t-BHP groups in comparison to the control group (2203.77 ± 194.05 vs. 368.87 ± 30.03, *p* < 0.001; 1449.93 ± 228.08 vs. 368.87 ± 30.03; 1549.2 ± 303.24 vs. 368.87 ± 30.03; 1457.58 ± 263.36 vs. 368.87 ± 30.03, *p* < 0.01). Significantly lower levels of this marker were found in the ME500, ME500+t-BHP, and RA+t-BHP groups compared to the S+t-BHP group (233.63 ± 18.79 vs. 2203.77 ± 194.05; 688.84 ± 65.7 vs. 2203.77 ± 194.05, *p* < 0.001; 1142.37 ± 260.22 vs. 2203.77 ± 194.05, *p* = 0.01).

As shown in Figure 2d, ALT levels were significantly higher in the S+t-BHP and ME250+t-BHP groups compared to the controls (390.06 ± 41.68 vs. 129.74 ± 10.28, *p* < 0.001; 310.46 ± 26.35 vs. 129.74 ± 10.28, *p* < 0.01). Conversely, ALT levels were significantly lower in the ME500, ME400+t-BHP, ME500+t-BHP, and Sil+t-BHP groups than in the S+t-BHP group (113.56 ± 16.3 vs. 390.06 ± 41.68, *p* < 0.001; 215.43 ± 31.67 vs. 390.06 ± 41.68; 207.44 ± 45.9 vs. 390.06 ± 41.68, *p* < 0.01; 242.60 ± 37.73 vs. 390.06 ± 41.68, *p* < 0.05).

Figure 2e demonstrates that urea levels increased significantly in the S+t-BHP, ME400+t-BHP, ME500+t-BHP, RA+t-BHP and Sil+t-BHP groups compared to the controls (4.71 ± 0.54 vs. 1.23 ± 0.14; 4.15 ± 0.56 vs. 1.23 ± 0.14; 4.76 ± 0.47 vs. 1.23 ± 0.14; 4.82 ± 0.33 vs. 1.23 ± 0.14; 5.28 ± 0.34 vs. 1.23 ± 0.14; *p* < 0.001). The ME500 group showed significantly lower urea levels than the S+t-BHP group (1.90 ± 0.52 vs. 4.71 ± 0.54, *p* ≤ 0.001).

Figure 2f exhibits notably elevated UA levels in the S+t-BHP group compared to the control group (734.69 ± 91.09 vs. 374.03 ± 21.03, *p* < 0.001). In addition, lower UA levels were observed in the ME500, ME400+t-BHP, ME500+t-BHP, RA+t-BHP and Sil+t-BHP groups compared to the S+t-BHP group (480.89 ± 68.6 vs. 734.69 ± 91.09, *p* ≤ 0.05; 337.29 ± 26.92 vs. 734.69 ± 91.09, *p* < 0.001; 470.98 ± 73.39 vs. 734.69 ± 91.09, *p* < 0.05; 436.55 ± 37.63 vs. 734.69 ± 91.09; 439.22 ± 27.29 vs. 734.69 ± 91.09, *p* ≤ 0.01).

Figure 2g demonstrates significantly lower Cr levels in the ME250+t-BHP, ME400+t-BHP, ME500+t-BHP, RA+t-BHP, and Sil+t-BHP groups in comparison to the S+t-BHP group (61.29 ± 1.85 vs. 76.53 ± 1.98; 59.52 ± 2.91 vs. 76.53 ± 1.98, *p* < 0.01; 58.07 ± 4.53 vs. 76.53 ± 1.98; 56.33 ± 2.28 vs. 76.53 ± 1.98; 57.39 ± 1.57 vs. 76.53 ± 1.98; *p* ≤ 0.001).

### 3.3. Assessment of Biomarkers of Oxidative Stress in the Liver Homogenate

**Figure 3:**
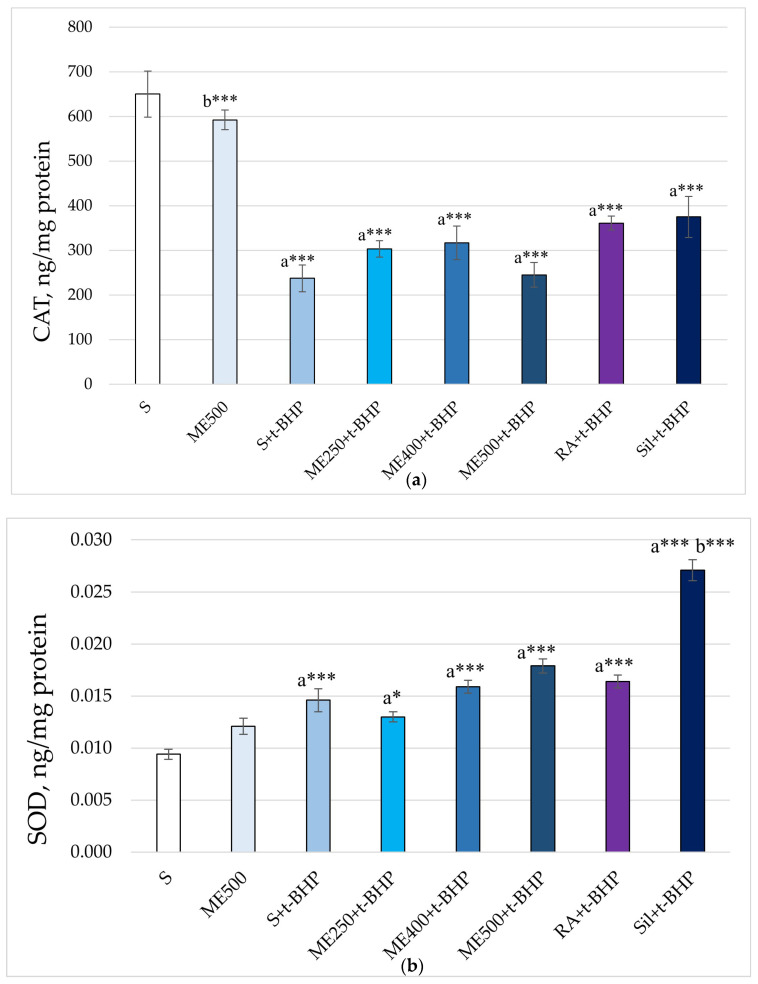

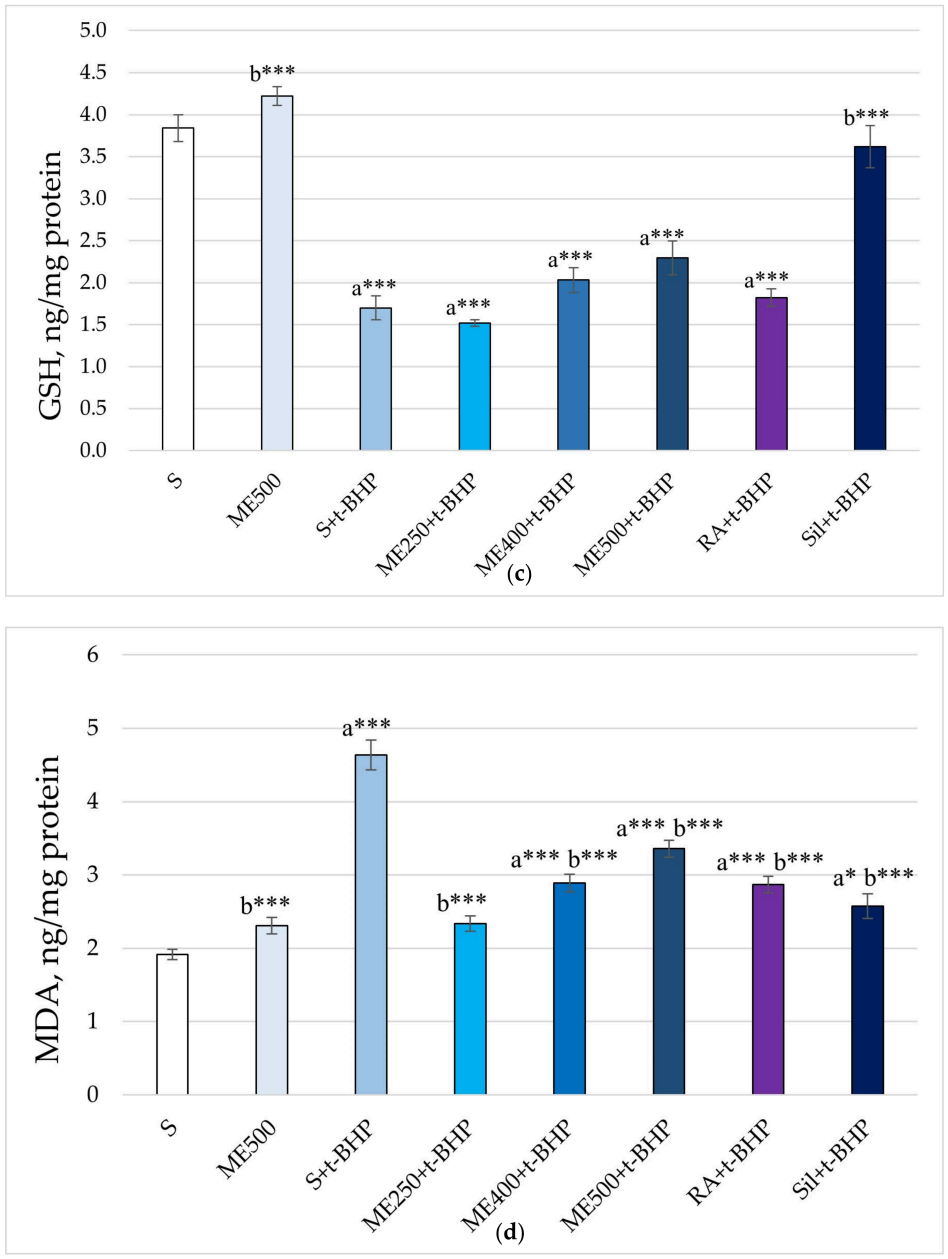

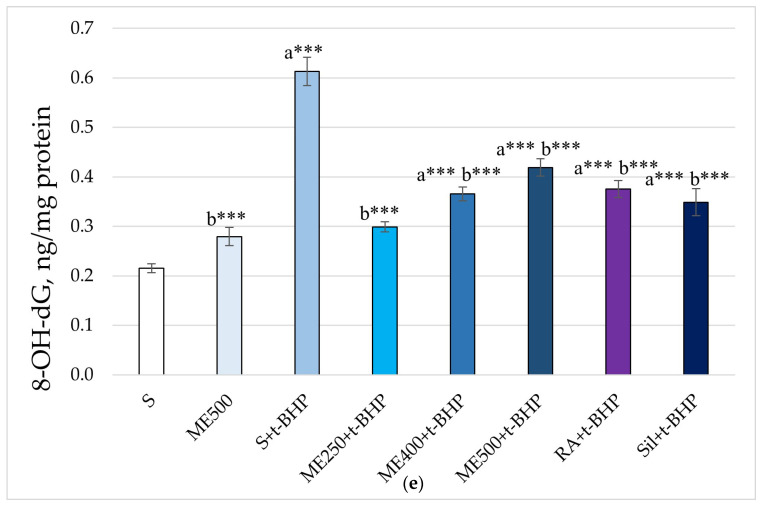
Biochemical markers in liver tissue. (**a**) CAT; (**b**) SOD; (**c**) GSH; (**d**) MDA; (**e**) 8-OHdG. Data are presented as mean ± SEM. The symbol a* indicates *p* < 0.05 vs. control group; a*** indicates *p* ≤ 0.001 vs. control group; b*** indicates *p* < 0.001 vs. t-BHP + saline-treated rats, according to one-way ANOVA followed by Tukey’s post hoc test.

Figure 3a shows significantly lower CAT levels in the S+t-BHP, ME250+t-BHP, ME400+t-BHP, ME500+t-BHP, RA+t-BHP, and Sil+t-BHP groups in comparison to the control group (237.36 ± 29.89 vs. 649.97 ± 51.64; 302.86 ± 18.53 vs. 649.97 ± 51.64; 316.80 ± 37.69 vs. 649.97 ± 51.64; 244.95 ± 27.69 vs. 649.97 ± 51.64; 360.91 ± 15.36 vs. 649.97 ± 51.64; 374.84 ± 45.86 vs. 649.97 ± 51.64, *p* < 0.001). On the other hand, a significant increase in CAT enzyme levels was observed in the group treated with ME500 compared to the S+t-BHP group (592.18 ± 21.66 vs. 237.36 ± 29.89, *p* < 0.001).

Significantly elevated SOD levels were observed in the S+t-BHP, ME250+t-BHP, ME400+t-BHP, ME500+t-BHP, RA+t-BHP, and Sil+t-BHP groups compared to the control group (0.015 ± 0.001 vs. 0.009 ± 0.0005, *p* < 0.001; 0.013 ± 0.0005 vs. 0.009 ± 0.0005, *p* < 0.05; 0.016 ± 0.0006 vs. 0.009 ± 0.0005; 0.018 ± 0.0007 vs. 0.009 ± 0.0005; 0.016 ± 0.0006 vs. 0.009 ± 0.0005; 0.027 ± 0.001 vs. 0.009 ± 0.0005, *p* < 0.001). Additionally, SOD levels were significantly higher in the Sil+t-BHP group in comparison to the S+t-BHP group (0.027 ± 0.001 vs. 0.015 ± 0.001, *p* < 0.001), as shown in Figure 3b.

Figure 3c demonstrates significantly lower GSH levels in the S+t-BHP, ME250+t-BHP, ME400+t-BHP, ME500+t-BHP, and RA+t-BHP groups in comparison to the control group (1.70 ± 0.14 vs. 3.84 ± 0.16; 1.52 ± 0.04 vs. 3.84 ± 0.16; 2.03 ± 0.15 vs. 3.84 ± 0.16; 2.29 ± 0.20 vs. 3.84 ± 0.16; 1.82 ± 0.10 vs. 3.84 ± 0.16, *p* < 0.001). Significantly increased levels of reduced GSH were observed in the ME500 and Sil+t-BHP groups compared to the S+t-BHP group (4.22 ± 0.11 vs. 1.70 ± 0.14; 3.62 ± 0.25 vs. 1.70 ± 0.14, *p* < 0.001).

As shown in Figure 3d, serum MDA levels increased significantly in the S+t-BHP, ME400+t-BHP, ME500+t-BHP, RA+t-BHP and Sil+t-BHP groups compared to the controls (4.63 ± 0.20 vs. 1.92 ± 0.07; 2.89 ± 0.12 vs. 1.92 ± 0.07; 3.36 ± 0.12 vs. 1.92 ± 0.07; 2.87 ± 0.11 vs. 1.92 ± 0.07, *p* < 0.001; 2.57 ± 0.17 vs. 1.92 ± 0.07, *p* < 0.05). There was a significant decrease in the ME500, ME250+t-BHP, ME400+t-BHP, ME500+t-BHP, RA+t-BHP and Sil+t-BHP groups compared to the S+t-BHP group (2.31 ± 0.11 vs. 4.63 ± 0.20; 2.33 ± 0.10 vs. 4.63 ± 0.20; 2.89 ± 0.12 vs. 4.63 ± 0.20; 3.36 ± 0.12 vs. 4.63 ± 0.20; 2.87 ± 0.11 vs. 4.63 ± 0.20; 2.58 ± 0.17 vs. 4.63 ± 0.20, *p* < 0.001).

Figure 3e illustrates a significant increase in 8-OHdG levels in the S+t-BHP, ME400+t-BHP, ME500+t-BHP, RA+t-BHP and Sil+t-BHP groups compared to the control rats (0.613 ± 0.029 vs. 0.215 ± 0.009; 0.366 ± 0.014 vs. 0.215 ± 0.009; 0.419 ± 0.018 vs. 0.215 ± 0.009; 0.375 ± 0.017 vs. 0.215 ± 0.009; 0.349 ± 0.027 vs. 0.215 ± 0.009, *p* < 0.001). Significantly lower levels of this marker were found in the ME500, ME250+t-BHP, ME400+t-BHP, ME500+t-BHP, RA+t-BHP, and Sil+t-BHP groups compared to the S+t-BHP group (0.279 ± 0.018 vs. 0.613 ± 0.029; 0.299 ± 0.01 vs. 0.613 ± 0.029; 0.366 ± 0.014 vs. 0.613 ± 0.029; 0.419 ± 0.018 vs. 0.613 ± 0.029; 0.375 ± 0.017 vs. 0.613 ± 0.029; 0.349 ± 0.027 vs. 0.613 ± 0.029, *p* < 0.001).

## 4. Discussion

Oxidative stress is closely linked to cellular damage and has been associated with the development of numerous pathological conditions [14]. The cytotoxicity of t-BHP is primarily mediated by the generation of free radical intermediates, which compromise cellular integrity. The latter also lead to the formation of covalent bonds with cellular macromolecules, resulting in DNA damage and subsequent cellular injury [13].

Alanine aminotransferase and AST are key biochemical indicators used to evaluate liver function, and elevated serum activities of these enzymes generally reflect hepatic injury. In this study, administration of t-BHP produced clear signs of liver toxicity, demonstrated by a marked rise in serum ALT and AST levels in treated rats, which corroborates earlier findings reported in the literature [40]. Since ALT is primarily cytosolic and AST is partly located in the cytosol, their leakage into the bloodstream indicates loss of membrane integrity in hepatocytes following structural damage to the liver tissue [15,40]. Notably, supplementation with 500 mg/kg of *M. frivaldszkyana* in t-BHP-exposed rats markedly lowered their abnormally high ALT and AST levels. This reduction may be associated with the ability of the plant’s phytochemical constituents to stabilise hepatocyte plasma membranes and support the repair of injured liver tissue, possibly through enhanced protein synthesis and stimulated hepatocyte regeneration [41].

Enhanced lipid peroxidation and free radical-derived alkoxyl and peroxyl species are believed to play a central role in t-BHP-induced hepatic damage [42]. In this study, lipid peroxidation and DNA damage were evaluated through measurements of MDA and 8-OHdG, with t-BHP administration markedly elevating the levels of both markers. Treatment with *M. frivaldszkyana* at all experimental doses significantly counteracted these increases. This protective activity is thought to stem from multiple antioxidant mechanisms, including interactions among its phenolic constituents, which may neutralise lipid peroxides and ROS, interrupt propagation of lipid peroxidation, and potentially inhibit CYP P450-mediated activation of t-BHP into reactive radicals [13,42]. On the other hand, the administration of the plant extract in all selected doses failed to significantly enhance the levels of the studied antioxidant enzymes as well as GSH. The latter underlies the fact that a reduction in oxidative damage markers such as MDA and/or 8-OHdG may occur independently of significant alterations in endogenous antioxidant enzymes. Hence, it could be suggested that the extract exerts its effects primarily through direct radical scavenging, inhibition of ROS generation, or interruption of lipid peroxidation pathways rather than through upregulation of enzymatic antioxidant defences. This notion is further supported by the registered radical scavenging activity of the studied plant species [27]. Furthermore, the absence of significant restoration of GSH levels following treatment with the plant extract may be explained by the extensive utilisation of glutathione during t-BHP detoxification. Since t-BHP metabolism directly consumes intracellular GSH, depletion of glutathione may persist despite attenuation of oxidative damage [43]. In contrast, silymarin is known to enhance endogenous antioxidant defences through stimulation of glutathione synthesis and activation of cytoprotective signalling pathways, which may explain its ability to significantly restore hepatic GSH levels [44].

Our earlier study of *M. frivaldszkyana* methanolic extract focused on its phytochemical characterisation. The CG-MS analysis showed that the primary metabolites included sugars and sugar alcohols (predominantly sucrose, glucose, mannose, fructose, maltose, galactinol, myo-inositol, and glycerol), organic acids (mainly citric and quinic acids), and amino acids, with proline and alanine being the most abundant. The lipidomic analysis revealed representatives of ten lipid classes, with triacylglycerols predominating. The secondary metabolite profile, determined by UPLC-MS-MS, was dominated by flavonoids, mainly flavonoid glycosides, including high levels of linarin and its derivatives, as well as chlorogenic acid, RA, rutin, and derivatives of quercetin, kaempferol, naringenin, and apigenin [28]. Many of these secondary metabolites have demonstrated hepatoprotective activity in experimental studies. Pretreatment with rutin has been shown to significantly mitigate t-BHP-induced liver damage in mice, as indicated by reduced levels of AST, ALT, lactate dehydrogenase, alkaline phosphatase, bilirubin, and albumin levels [45]. Linarin pretreatment markedly lowered ALT, AST, and total bilirubin concentrations in a CCl_4_-induced hepatic toxicity model [46]. The protective effects of apigenin against t-BHP-induced oxidative stress in ARPE-19 cells are attributed to its antioxidant activity via activation of Nrf2 signalling pathway [47]. Quercetin, the major flavonoid in *Capparis spinosa*, ameliorated t-BHP-induced liver injury in mice, as evidenced by reduced serum enzyme markers and lower MDA levels, and confirmed by histopathology. These effects are likely via antioxidant and radical scavenging effects [48]. Similarly, chlorogenic acid and cynarin, the main phenolic acids in aqueous artichoke leaf extracts, reduced MDA formation in t-BHP-exposed rat hepatocytes [49]. Zhang et al. (2018) reported that oral treatment with RA-rich extract from cold-pressed *Perilla frutescens* seed flour markedly reduced t-BHP-induced increases in AST and ALT levels, along with hepatocyte degeneration and neutrophilic infiltration [50]. The cellular antioxidant effects of *Salvia officinalis* (*S.officinalis*), including the inhibition of t-BHP-induced cell death and lipid peroxidation in HepG2 cells, are closely associated with its major phenolic constituents, namely RA and luteolin-7-glucoside [51].

The kidneys play a key role in maintaining homeostasis by regulating body fluid volume and electrolytes, controlling hormone release and blood pressure, and eliminating toxic metabolites [52]. Urea, Cr, and UA are widely employed clinical biomarkers of renal function, and their serum levels increase in response to renal impairment [31]. Accordingly, in the current study, t-BHP exposure caused an increase in serum urea, Cr, and UA levels in rats, indicative of compromised kidney function, which is consistent with published research [53].

The present results show that methanolic extract of *M. frivaldszkyana* lowered UA levels across all tested doses, with the most pronounced effect observed at 400 mg/kg, followed by the highest dose. These findings align with studies showing protective effects of polyphenols against t-BHP-induced oxidative stress. In particular, supplementation with *Aspalathus linearis* (rooibos), which is rich in phenolic compounds such as rutin, has been shown to significantly reduce hepatic MDA levels and serum enzyme concentrations, thereby mitigating t-BHP-induced tissue injury, which is typically characterised by increased MDA, as well as serum ALT, AST, and UA levels [53].

In our investigation, markedly lower UA levels were also noted in the groups treated with silymarin and RA. Furthermore, treatment with the methanolic extract of *M. frivaldszkyana* was associated with a notable dose-dependent attenuation of Cr levels, while silymarin and RA also produced significant reductions in Cr levels.

To our knowledge, there are no published data on the role of RA in t-BHP-induced renal injury. Nevertheless, RA has been shown to exert nephroprotective effects in other experimental models. Rosmarinic acid mitigated methotrexate-induced renal and hepatic toxicity by reducing elevations in urea, Cr, ALT, AST, and lipid peroxidation while restoring antioxidant defences, reflecting its antioxidant activities [54]. The oral administration of 200 mg/kg cold-pressed *Rosmarinus officinalis* oil for 8 weeks mitigated carbon tetrachloride-induced (CCl_4_) liver toxicity in rats. This was demonstrated by reductions in serum urea, UA, Cr, ALT, and AST levels, as well as hepatic MDA, indicating hepatoprotective and nephroprotective effects [55]. Marked increases in serum urea, UA, and Cr levels were shown in rats exposed to chlorpyrifos or methomyl compared to controls. These elevations were significantly attenuated by treatment with ethanolic extracts of *S. officinalis* and *Ruta graveolens* (*R. graveolens*), indicating protection against pesticide-induced renal toxicity [56]. Rosmarinic acid is the most abundant polyphenol found in *S. officinalis* infusion extract, followed by rutin, chlorogenic acid, and quercetin [57]. Rutin was the predominant compound identified in *R. graveolens*, with naringenin present at lower levels [58]. Naringin has been widely recognised for its protective effects in numerous oxidative stress models, attributed to its capacity to neutralise free radicals directly [59]. Other individual constituents present in *M. frivaldszkyana* also exhibit renoprotective effects. For instance, chlorogenic acid has been shown to attenuate oxidative, inflammatory, and apoptotic damage in tamoxifen-induced liver and kidney toxicity [60]. Linarin, a glycosylated flavone found among plants of the Asteraceae and Lamiaceae families, has been reported to mitigate cisplatin-induced renal toxicity through its anti-inflammatory effects and ability to suppress oxidative stress [61]. A pharmacokinetic study indicates that linarin is rapidly absorbed in rats [62]. Accordingly, it can be hypothesised that the observed antioxidant activity is partially attributable to the high concentration of linarin within the extract, although this effect is more likely to be the result of the full range of compounds present. This observation is consistent with the widely accepted concept that the biological activity of plant extracts is determined by the combined effect of multiple constituents rather than by a single compound or the constituent with the highest individual biological activity [63].

Based on the results presented, we can suggest that pretreatment with the methanolic extract of *M. frivaldszkyana* may have beneficial effects on t-BHP-induced liver injury and may improve serum biomarkers associated with renal dysfunction. The hepatoprotective effect could be related to cell membrane protection, decreased lipid peroxidation and DNA damage, and improved tissue regeneration. The mechanism by which it benefits kidney function remains unknown and is within the scope of our future research. The extract’s antioxidant in vivo properties provide a basis for investigating its effectiveness against other animal models of toxicity.

The current study has the following limitations: t-BHP-induced toxicity was evaluated only in male Wistar rats. Therefore, the results may differ for female animals, other rat strains, or different routes of administration. The pretreatment period was five days prior to t-BHP application; a longer pretreatment period may yield a different outcome. In addition, t-BHP-induced toxicity was evoked by a single application of the agent; thus, chronic exposure may influence the observed results. Another limitation is the lack of extract standardisation and residual solvent control. Finally, the study focused primarily on biochemical and histological evaluations, rather than molecular signalling pathways. As a result, the exact intracellular mechanisms responsible for the observed protective effects remain to be elucidated in future investigations.

## 5. Conclusions

Pretreatment with the methanolic extract of *M. frivaldszkyana* may have beneficial effects on t-BHP-induced liver injury and attenuate alterations in serum renal function biomarkers in male Wistar rats. Cell membrane protection, reduced lipid peroxidation and DNA damage, and improved tissue regeneration may be related to its hepatoprotective effect. However, the present study did not include renal histopathology or assessment of oxidative stress markers in kidney tissue; therefore, any renoprotective effect remains preliminary and requires further investigation. The high content of linarin, RA, chlorogenic acid, and rutin in the extract may contribute to the improvement in serum renal biomarkers observed in this study. The extract’s antioxidant properties may play a critical role in the recorded effects.

## Figures and Tables

**Table 1:** Roenigk classification (adopted from Berends et al. (2007) [37]).

Grade	Accumulation of Fats	Nuclear Pleomorphism	Fibrosis	Necrosis/Inflammation
1	Mild or none	Mild or none	None	With or without mild portal inflammation
2	Moderate or severe	Moderate or severe	None	Moderate or severe portal inflammation
3a	With or without	With or without	Mild (fibrosis extending into acini)	With or without
3b	With or without	With or without	Moderate or severe	With or without
4	With or without	With or without	Cirrhosis	With or without

## Data Availability

The data presented in this study are available on request from the corresponding author.

## Abbreviations

The following abbreviations are used in this manuscript:

8-OHdG	8-hydroxy-2′-deoxyguanosine
ALT	Alanine aminotransferase
AST	Aspartate aminotransferase
CAT	Catalase
CCl_4_	Carbon tetrachloride
Cr	Creatinine
CYP	Cytochrome P450
DNA	Deoxyribonucleic acid
ELISA	Enzyme-linked immunosorbent assay
GPx	Glutathione peroxidase
GSH	Glutathione
MDA	Malondialdehyde
ORAC	Oxygen radical absorbance capacity
RA	Rosmarinic acid
ROS	Reactive oxygen species
SOD	Superoxide dismutase
t-BHP	Tert-butyl hydroperoxide
UA	Uric acid
